# Long noncoding RNA cytoskeleton regulator RNA promotes cell invasion and metastasis by titrating miR‐613 to regulate ANXA2 in nasopharyngeal carcinoma

**DOI:** 10.1002/cam4.2778

**Published:** 2019-12-20

**Authors:** Wei Chen, Mingyu Du, Xinyu Hu, Hongxia Ma, Erbao Zhang, Tingting Wang, Li Yin, Xia He, Zhibin Hu

**Affiliations:** ^1^ The Affiliated Cancer Hospital of Nanjing Medical University Jiangsu Cancer Hospital Jiangsu Institute of Cancer Research Nanjing Jiangsu China; ^2^ Jiangsu Key Lab of Cancer Biomarkers, Prevention and Treatment Department of Epidemiology Collaborative Innovation Center For Cancer Personalized Medicine School of Public Health Nanjing Medical University Nanjing China

**Keywords:** CYTOR, metastasis, nasopharyngeal carcinoma

## Abstract

**Background:**

Nasopharyngeal carcinoma (NPC) is one of the most frequent head and neck malignant tumors. Long noncoding RNAs play critical roles in tumorigenesis.

**Methods:**

Real‐time quantitative PCR arrays were used to evaluate the expression levels of cytoskeleton regulator RNA (CYTOR) in NPC tissues and cells. Cell counting kit‐8 and colony formation analyses were used to test the NPC cell viability, while wound healing and transwell assays were employed to detect cell invasion and migration ability. Luciferase reporter assay and Western blot analyses were employed to explore the relationships among CYTOR, miR‐613, and ANXA2.

**Results:**

We found that CYTOR expression was elevated both in NPC tissues and cells. Functional assays revealed that CYTOR promoted the invasion and migration of NPC cells. The established spontaneous lymph node metastasis model also confirmed that CYTOR promoted NPC cell metastasis in vivo. Mechanically, we found that the subcellular localization of CYTOR mostly occurred in the cell cytoplasm. Luciferase reporter and RIP assays confirmed that CYTOR functioned as the molecular sponge of miR‐613. Subsequent experiments confirmed that ANXA2 was directly targeted by miR‐613. Gain‐ and loss‐of‐function studies further confirmed that CYTOR induced the upregulation of ANXA2 by competitively binding to miR‐613, thus leading to NPC metastasis.

**Conclusion:**

Our results highlight the importance of CYTOR in NPC development and provide new insights into potential therapeutic targets for NPC.

## Graphical abstract

Cytoskeleton regulator RNA (CYTOR) is upregulated in nasopharyngeal carcinoma (NPC) tissues and cells and facilitates the invasion and metastasis of NPC cells. CYTOR induced the upregulation of ANXA2 by competitively binding to miR‐613, thus leading to NPC metastasis.

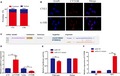

## 1 INTRODUCTION

Nasopharyngeal carcinoma (NPC) is a malignant tumor originating from nasopharyngeal epithelial cells, and 80% of new global cases occur in Asia.^1^, ^2^ The number of newly diagnosed NPC patients in China is approximately 60 600 per year, accounting for 40% of global cases, and it is highly prevalent in Guangdong and Guangxi Province.^3^ Distant metastasis and recurrence remain the main model for the treatment failure of NPC.^1^, ^2^ Therefore, revealing the underlying mechanism of invasion and migration of NPC is necessary for developing therapeutic strategies.

Long noncoding RNAs (lncRNAs) are RNA transcripts that are longer than 200 nucleotides without protein‐coding potential, which lack open reading frames.^4^ lncRNAs act as signals, guides, scaffolds, or decoys in different biological molecules, regulate a wide range of biological processes, and alter gene expression via transcriptional, posttranscriptional, and epigenetic modifications.^5^ Aberrant lncRNA expression plays critical roles in tumorigenesis.^6^ LncRNA cytoskeleton regulator RNA (CYTOR), also known as LINC00152, which is located in chromosome region 2p11.2, is overexpressed in many cancers.^7^, ^8^, ^9^ However, the expression levels of lncRNA CYTOR and its biological functions and molecular pathogenesis in NPC remain largely ambiguous.

In 2011, Salmena et al proposed the competing endogenous RNAs (ceRNA) hypothesis, a competitive endogenous RNA mechanism for posttranscriptional regulation, which competes with microRNA by sharing more than one microRNA response element (MRE).^10^ lncRNA can sponge microRNAs, thereby eliminating the inhibition of microRNAs on its target genes, thus affecting the function of microRNAs and increasing the expression level of target genes.^11^, ^12^

This work aims to explore the expression of CYTOR in NPC samples and cells. We further investigated the biological functions and the underlying mechanism in regulating the tumorigenesis of NPC. Our results highlight the importance of CYTOR in NPC development and provide new insights into potential therapeutic targets for the treatment of NPC.

## 2 MATERIALS AND METHODS

### 2.1 Clinical specimens and cell culture

Five human NPC cell lines (CNE‐1, CNE‐2, 5‐8F, SUNE‐1, and 6‐10B) were maintained in RPMI‐1640 (Corning) supplemented with 5% fetal bovine serum (Gibco). The human immortalized nasopharyngeal epithelial cell line (NP69) was cultivated in keratinocyte/serum‐free medium covered in growth factors (Gibco). Both materials were provided by the American Tissue Culture Collection and were grown in a humidified incubator of 5% CO_2_ at 37°C. Seventeen NPC tissues and five normal healthy nasopharyngeal tissues, which were obtained from Jiangsu Cancer Hospital, were stored in a laboratory freezer at −80°C. The diagnosis of all specimens was histopathologically confirmed by a pathologist. This study was approved by the Institutional Ethical Review Board of Jiangsu Cancer Hospital, and each patient provided written informed consent.

RNA extraction, reverse transcription, and quantitative polymerase chain reaction (RT‐qPCR).

TRIzol reagent (Invitrogen) was used to extract the total RNA from NPC cell lines or clinical samples following the manufacturer's instructions. RNAs were reverse transcribed into complementary DNA (cDNA) using Moloney murine leukemia virus reverse transcriptase (Promega). Real‐time PCR was employed using the 2^−∆∆CT^ method at a total volume of 20 μL, including 10 µL of SYBR Green PCR Master Mix (Invitrogen), 1 μL of each F or T primer, 4 μL of DEPC water, and 5 μL of cDNA. The control group was normalized to the small nuclear RNA RNU6B (U6 snRNA) and β‐actin expression level. The sequences of specific primers used are as follows: CYTOR: 5′‐TTGATGGCTTGAACATTTGG‐3′ and 5′‐TCGTGATTTTCGGTGTCTGT‐3′; ANXA2: 5′‐TCTACTGTTCACGAAATCCTGTG‐3′ and 5′‐AGTATAGGCTTTGACAGACCCAT‐3′; β‐actin: 5′‐GGACTTCGAGCAAGAGATGG‐3′ and 5′‐AGCACTGTGTTGGCGTACAG‐3′.

### 2.2 Western blot analysis

Total proteins were isolated from the transfected cell lines using the RIPA buffer, phosphatase inhibitor, and PMSF (Beyotime), and its concentration was quantified using the BCA protein assay kit (Beyotime) following the manufacturer's protocols. All proteins were subsequently separated via sodium dodecyl sulfate‐polyacrylamide gel electrophoresis and transferred onto polyvinylidene fluoride membranes (Millipore). After blocking with bovine serum albumin, the membranes were embraced overnight with the primary and secondary antibodies. The following primary antibodies were employed: anti‐β‐actin (1:1,000, Cell Signaling Technology); anti‐ANXA2 (1:1,000, Cell Signaling Technology). Immunoreactive bands were visualized using the ECL detection reagent (Millipore).

### 2.3 Cell transfection and generation of stably transfected cell line

Smart silencer targeting CYTOR and negative control were purchased from RiboBio. For CYTOR overexpression studies, 6‐10B cells were infected with lentivirus containing the CYTOR‐GV367 plasmid synthesized by GeneChem (Shanghai, China). The full length of ANXA2 was synthesized by Genecreate. The short hairpin RNA (siRNA) targeting ANXA2 was obtained from RiboBio. The abovementioned siRNAs and plasmids were transfected using Lipofectamine 2000 according to the manufacturer's instructions.

### 2.4 Colony formation assay

CNE‐1 and 6‐10B cells were placed in six‐well plates (1 × 10^3^ cells/well) after transfection and then incubated at 37°C in 5% CO_2_. After 14 days, cells were washed with phosphate buffer saline (PBS), fixed with paraformaldehyde overnight, and dyed with 0.4% crystal violet for 30 minute. The colonies were counted under an inverted microscope. Each cell line had triplicate wells.

### 2.5 Cell counting kit‐8 assay

Cell proliferation was monitored using cell counting kit‐8 (CCK8) assay (Promega) every 24 hour according to the manufacturer's protocol. After transfection, the cells were grown in 96‐well plates (3000 cells/well). Exactly 10 μL of CCK8 was added into each well. Cellular viability was detected via spectrophotometry at 450 nm after being cultured for 1 hour. This experiment was performed in five reduplicative wells, and the results were all averaged.

### 2.6 Wound healing assay

Transfected CNE‐1 and 6‐10B cells were seeded in 6‐well plates and allowed to reach 80%‐90% confluence cultivated with serum starvation for 24 hour. The cell monolayers were wounded with a 200‐µL RNase‐free pipette tube. The width of the scratch was recorded at 0 and 24 hour using an optical microscope to measure the percentage of the area covered by the migrated cells.

### 2.7 Transwell migration and invasion assay

NPC cell invasion and migration were determined using transwell chambers (Corning) without or with Matrigel (BD Biosciences). After being cultured with serum starvation for 24 hour, the transfected cells that were resuspended (2 × 10^4^ cells per well) in 200 µL of serum‐free medium were plated in the upper chambers. The lower chambers were added with 20% FBS and 500 µL of RPMI‐1640. After 24 or 36 hour of incubation, the membranes were fixed with paraformaldehyde overnight, stained with 0.1% crystal violet, and imaged. The invaded cells were counted using an inverted microscope.

### 2.8 Subcellular fractionation location

The RNA isolation of nuclear and cytoplasmic fractions generally followed the PARIS Kit (Invitrogen) according to the manufacturer's protocol. For fluorescence in situ hybridization (FISH), CNE‐1 and 6‐10B cells were fixed in 4% formaldehyde for 20 minute and subsequently permeabilized with 0.5% Triton X‐100 at 4°C. Hybridization buffer was supplemented with FISH probe incubated for 4 hour at 55°C, and cells were washed with 2 × saline‐sodium citrate (5‐6 times). Signal was detected by horseradish peroxidase‐conjugated anti‐DIG secondary antibodies (Jackson). After nucleus staining by DAPI, images were captured using an Olympus confocal laser scanning microscope.

### 2.9 Dual‐luciferase reporter assays

Dual‐luciferase reporter assay was performed to identify the relationships between lncRNA CYTOR and miR‐613. Wild‐type lncRNA CYTOR, mutant lncRNA CYTOR, wild‐type ANXA2 (WT‐3′UTR‐ANXA2), and mutant ANXA2 (MUT‐3′UTR‐ANXA2) were co‐transfected with miR‐613 mimic or miR‐613 mimic control into cells. After 48 hour, the relative luciferase activities in each group were measured using the dual‐luciferase reporter assay system (Promega) according to the manufacturer's instructions.

### 2.10 RIP

RIP assay was applied using the EZMagna RIP kit (Millipore) according to the manufacturer's instructions. Anti‐argonaute‐2 (Ago2) (ab32381; Abcam) and anti‐IgG antibodies were used for immunoprecipitation. qRT‐PCR assay was used to detect the enrichment of CYTOR and miR‐613 to Ago2.

### 2.11 In vivo nude mouse xenograft tumor models

Ten immunodeficient male BALB/c nude mice (6‐8 weeks old) were purchased from Nanjing Medical University Medical Center. To establish the spontaneous lymph node metastasis model, we separately injected 2 × 10^6^ stably transfected 6‐10B cells expressing CYTOR or control in 20 µL of cell suspension into the foot pad of each mouse. At 6 weeks after injection, all mice were euthanized, and the presence of axillary lymph node metastasis was observed via autopsy. Tumor metastasis experiment was performed under the approval of the Animal Science Committee of the Animal Science of Nanjing, China.

### 2.12 Statistical analysis

Statistical analyses were performed using SPSS 20.0 (SPSS) and GraphPad Prism 6 (GraphPad) software to assess significant differences in measured variables among groups. Data were presented as the means ± standard error mean and calculated using two‐way ANOVA, Student's *t* test, and paired *t* test. Significance was considered at *P* < .05 (*P < .05, **P < .01).

## 3 RESULTS

### 3.1 CYTOR expression was upregulated in NPC tissues and cells

**Figure 1:**
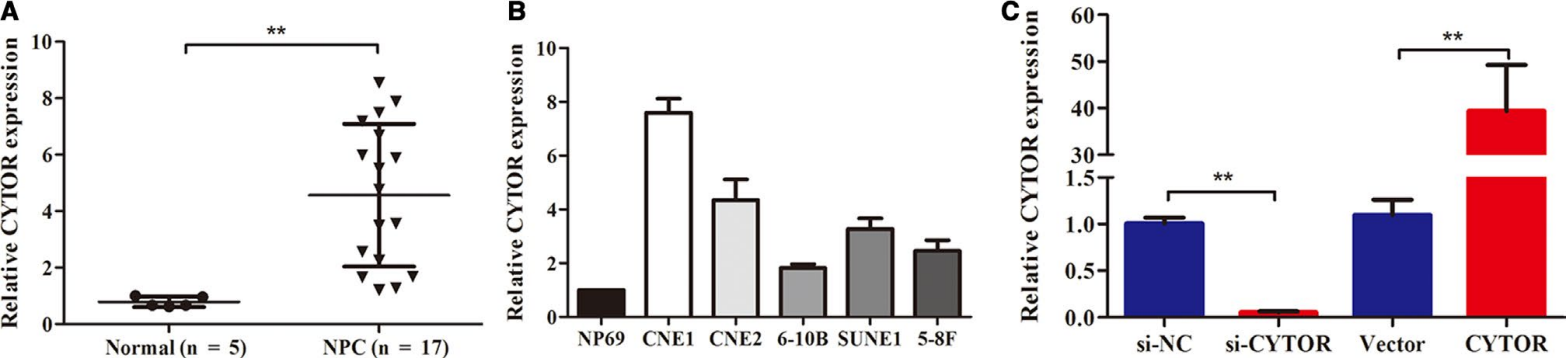
CYTOR expression was upregulated in NPC tissues and cells. A,B, The expression levels of CYTOR were detected with qRT‐PCR in NPC tissues and cells. C, The efficiencies of the interference and overexpression for CYTOR were determined via qRT‐PCR. *P < .05, **P < .01. Abbreviations: CYTOR, cytoskeleton regulator RNA; NPC, nasopharyngeal carcinoma

To comprehensively characterize the expression of CYTOR in NPC, we measured the CYTOR expression levels in 17 NPC tissues and five normal healthy nasopharyngeal tissues. According to the results, we found that CYTOR expression showed higher levels in NPC tissues than in normal controls (Figure 1A). Subsequently, the expression levels of CYTOR in five NPC cell lines and immortalized NP69 cells were evaluated via qRT‐PCR. As shown in Figure 1B, the expression of CYTOR was apparently upregulated in NPC cells compared with the controls.

### 3.2 CYTOR facilitated the invasion and migration of NPC cells

**Figure 2:**
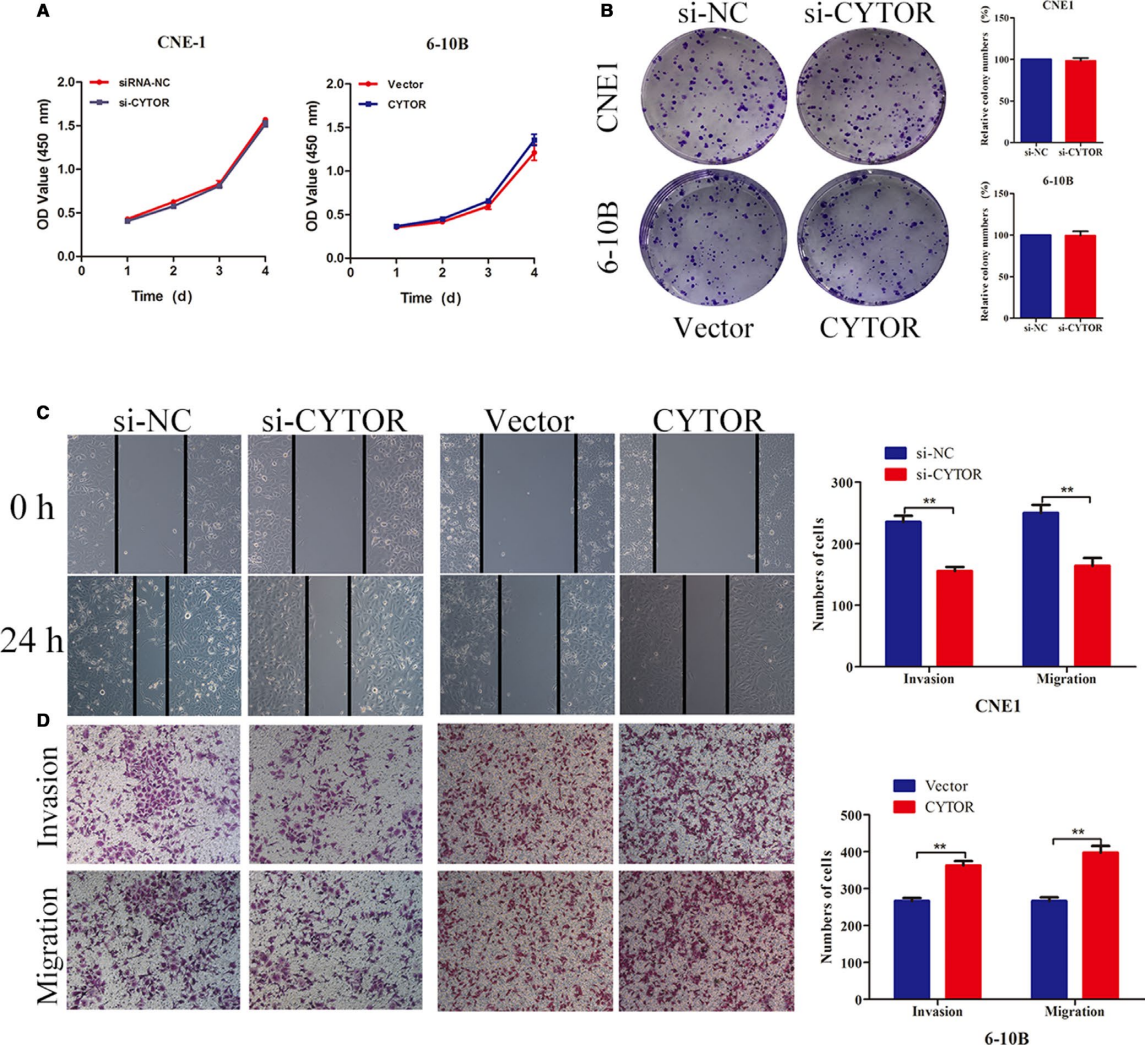
CYTOR facilitated the invasion and migration of NPC cells. A, CCK8 assays were used to detect the cell viability of NPC cells. B, Colony formation was applied to test the cell proliferation. C, Wound healing analyses were employed to explore the cell migration. D, Cell invasion and migration were detected by transwell analyses. *P < .05, **P < .01. Abbreviations: CCK8, cell counting kit‐8; CYTOR, cytoskeleton regulator RNA; NPC, nasopharyngeal carcinoma

**Figure 3:**
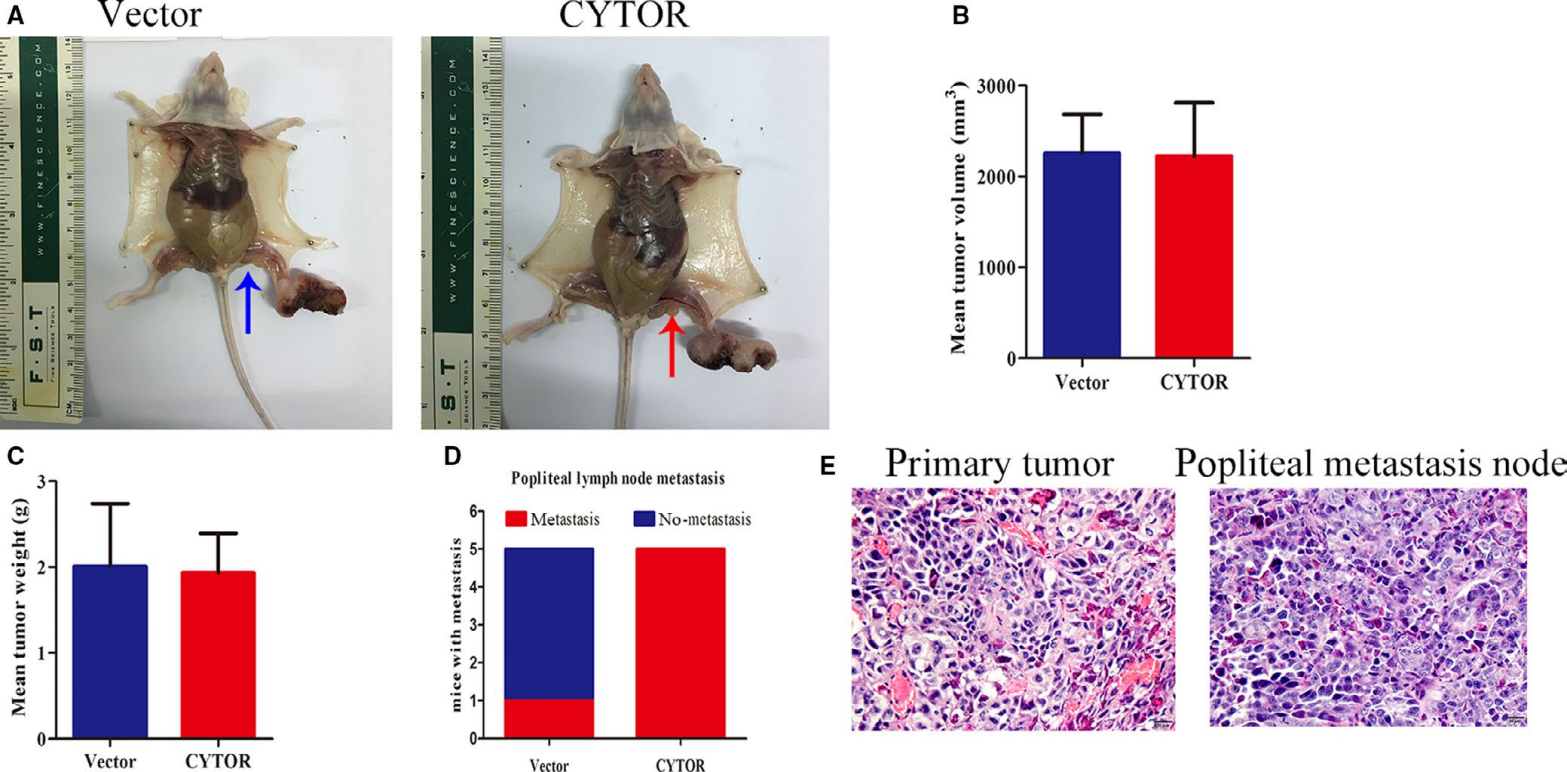
CYTOR facilitated the metastasis of NPC cells in vivo. A, Representative images of the presence of popliteal lymph node metastasis after necropsy in different groups. B, Tumor volume averages between the vector and CYTOR groups. C, Tumor weight averages between the vector and CYTOR groups. D, Incidences of metastasis in mice that received footpad injections of each group. E, H&E staining of the primary tumor and paired popliteal metastatic node. Abbreviations: CYTOR, cytoskeleton regulator RNA; H&E, hematoxylin and eosin; NPC, nasopharyngeal carcinoma

To further explore the biological roles of CYTOR in NPC carcinogenesis, we first transfected CNE1 cells with the highest expression of CYTOR with specific siRNA. We construct a vector containing the full‐length CYTOR transcript and transfected 6‐10B cells, which lack endogenous CYTOR expression. RT‐qPCR results implied that the transfection models were successfully established (Figure 1C). Functionally, CCK8 assays showed that CYTOR slightly affected the proliferation of NPC cells (Figure 2A). In agreement with the results of CCK8, no significant effect was observed in the colony formation between the treat groups and the controls (Figure 2B). Interestingly, the results of wound healing and transwell invasion assays showed that CYTOR knockdown weakened the invasion and migration capacities of NPC cells, whereas the reintroduction of CYTOR showed the opposite phenomenon (Figure 2C,D). To appraise metastatic potential in vivo, we established the spontaneous lymph node metastasis model, which was reported in our previous work. The experimental results illustrated that CYTOR overexpression promoted the tumor axillary lymph node metastasis (Figure 3A‐C).

These results reveal that CYTOR facilitated the invasive and migratory capabilities of NPC cells in vitro and vivo but did not significantly affect the cell proliferation.

### 3.3 CYTOR was the target of miR‐613

**Figure 4:**
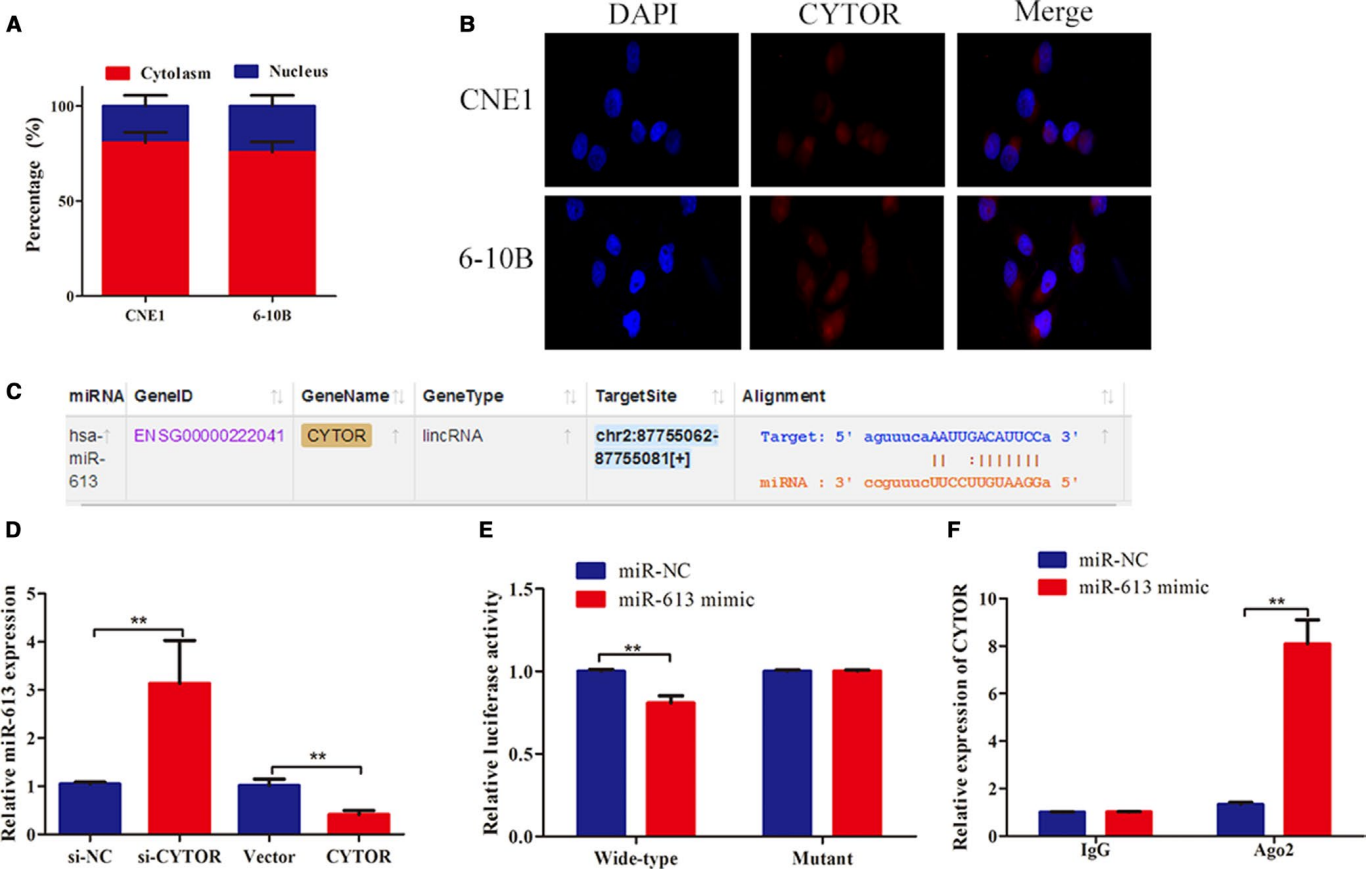
CYTOR was the target of miR‐613. A,B, The results of nuclear mass separation and FISH assays indicated that the majority of CYTOR was located in the cytoplasm. C, Schematic of the sequences of the predicted miR‐613 binding site in the 3′‐UTR of CYTOR. D, qRT‐PCR illustrated that CYTOR knockdown increased the expression of miR‐613, while CYTOR overexpression suppressed the expressions. E, Luciferase reporter assays were employed to test the luciferase activity in NPC cells. F, The association between CYTOR and miR‐613 was determined in NPC cells using RIP with the Ago2 antibody. *P < .05, **P < .01. Abbreviations: CYTOR, cytoskeleton regulator RNA; FISH, fluorescence in situ hybridization

**Figure 5:**
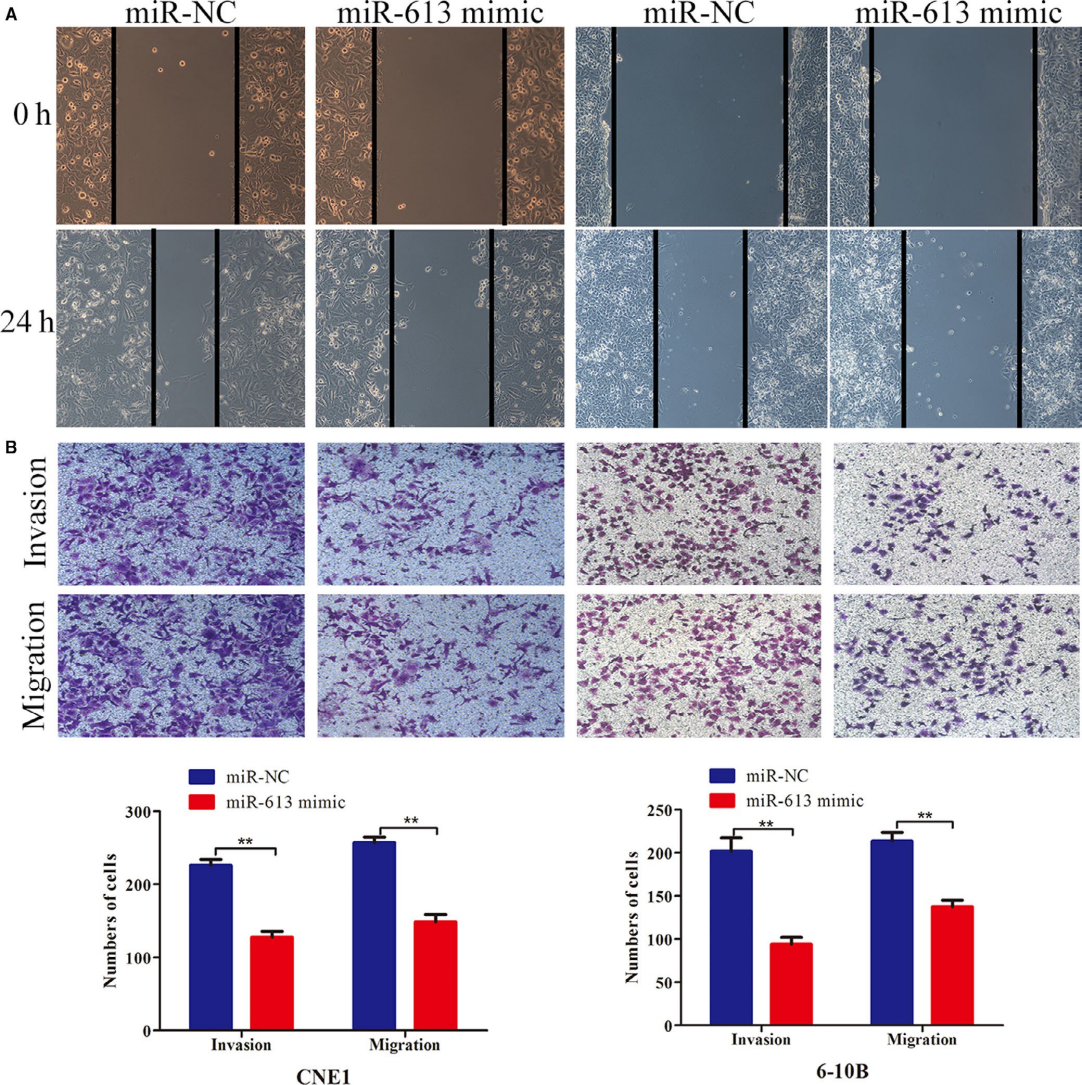
miR‐613 inhibited the invasion and migration of NPC cells. A,B, Wound healing and transwell assays showed that miR‐613 overexpression suppressed cell invasion and migration. *P < .05, **P < .01. Abbreviation: NPC, nasopharyngeal carcinoma

To accurately characterize the mechanistic basis for the biological functions of CYTOR, we employed subcellular fractionation assays and RNA FISH. The results displayed that CYTOR was predominantly distributed in the cell cytoplasm instead of the nucleus, indicating that CYTOR performs its regulatory functions at the posttranscriptional level (Figure 4A,B). Hence, CYTOR might function as the molecular sponge by affecting the expression of miRNAs. Online software program Starbase 3.0 (http://starbase.sysu.edu.cn) was utilized to predict the conjectural miRNA binding and found that CYTOR formed a complementary base pairing with miR‐613 (Figure 4C). We therefore employed qRT‐PCR to determine whether miR‐613 could directly target CYTOR. As illustrated in Figure 4D, CYTOR knockdown significantly raised the expression of miR‐613, whereas CYTOR overexpression downregulated the expression of miR‐613. Furthermore, luciferase reporter analyses illustrated that miR‐613 mimic exerted inhibitory effects on the luciferase activities of the wild‐type 3′‐UTR of CYTOR, but showed no obvious effects on the luciferase activity of the mutant (Figure 4E). RIP experiments also confirmed that CYTOR and miR‐613 were both enriched in microribonucleoproteins containing AGO2 compared with the control IgG (Figure 4F). A few works had demonstrated the role of miR‐613 in NPC, and some cellular behavioral experiments have been conducted to explore the effects of miR‐613 on the invasion and migration of NPC cells. The wound healing and transwell assays visualized that the restoration of miR‐613 tremendously compromised the NPC cells to migrate and invade (Figure 5A,B).

### 3.4 CYTOR promoted the malignant phenotypes of NPC cells via the repression of miR‐613

**Figure 6:**
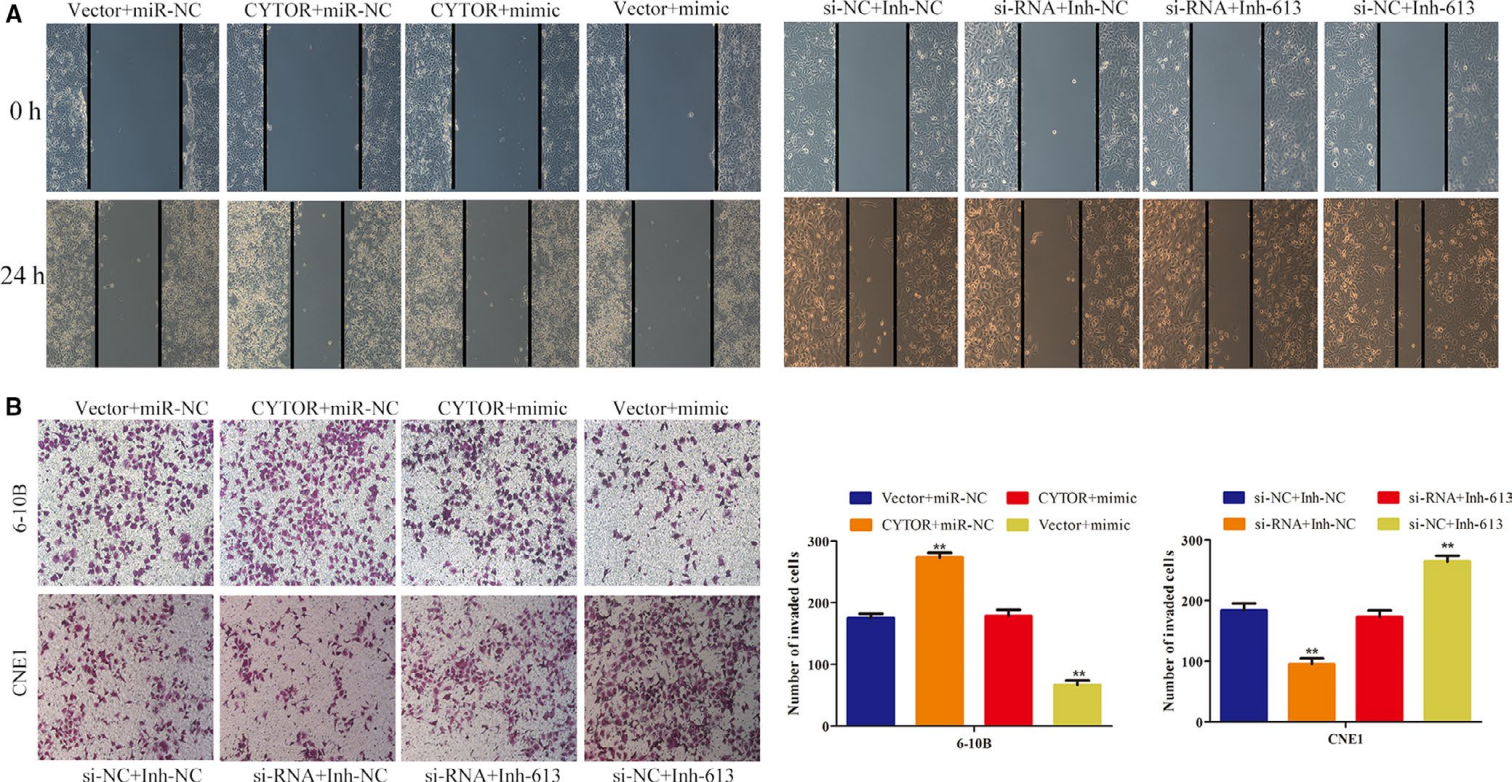
CYTOR promoted the malignant phenotypes of NPC cells via the repression of miR‐613. A,B, Wound healing and transwell assays indicated that CYTOR promoted the malignant phenotypes of NPC cells via the repression of miR‐613. *P < .05, **P < .01. Abbreviations: CYTOR, cytoskeleton regulator RNA; NPC, nasopharyngeal carcinoma

We employed rescue experiments to investigate whether the positive effects of CYTOR were mediated by miR‐613. We transfected overexpressed CYTOR cells with miR‐613 mimic and observed that the ectopic expression of miR‐613 attenuated the enhancement of cell invasion and migration, which were induced by CYTOR overexpression (Figure 6A,B). Moreover, we transfected CNE1 cells with si‐CYTOR after transfection of miR‐613 inhibitor and found that miR‐613 knockdown reversed the CYTOR knockdown‐mediated suppression of the invasion and migration of NPC cells (Figure 6A,B). Taken together, our data confirmed that CYTOR promoted the NPC cell invasion and migration by downregulating miR‐613 expression.

### 3.5 CYTOR promoted the invasion and migration of NPC cells via the miR‐613‐ANXA2 axis

**Figure 7:**
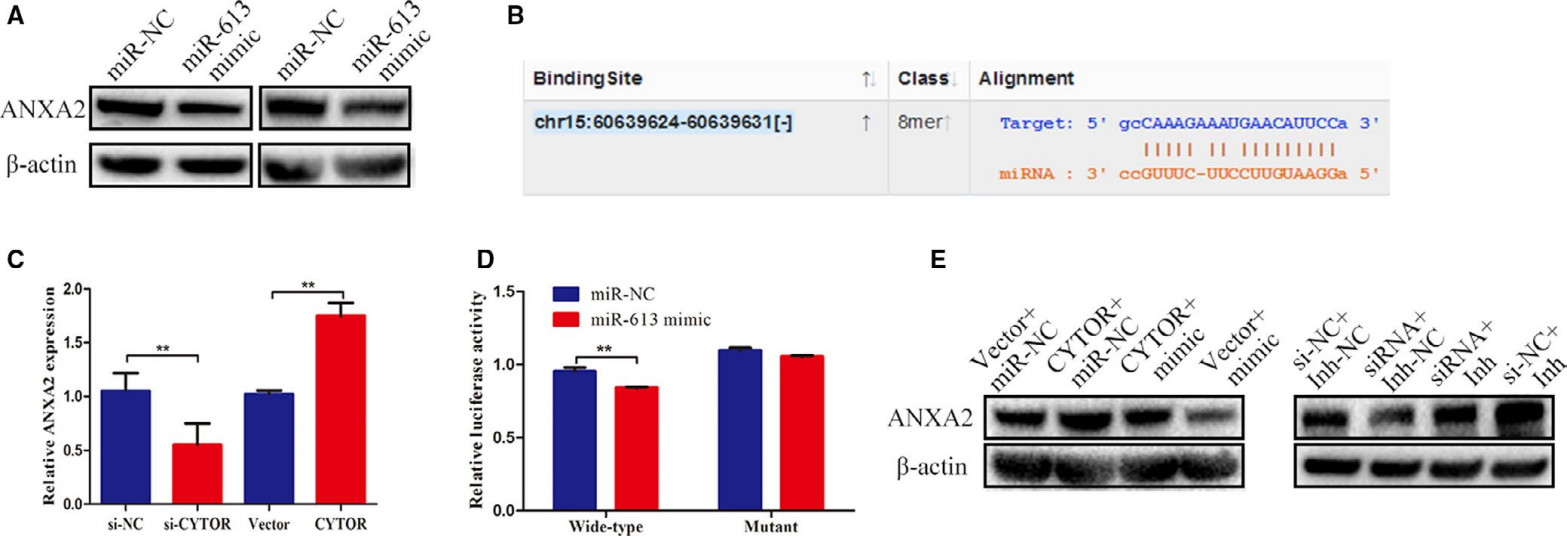
CYTOR promoted the expression of ANXA2 via the repression of miR‐613. A, The results of Western blot analyses showed that miR‐613 overexpression inhibited the expression of ANXA2. B, Schematic of the sequences of the predicted miR‐613 binding site in the 3′‐UTR of ANXA2. C, qRT‐PCR results exhibited that CYTOR knockdown repressed the expression of miR‐613, whereas CYTOR overexpression promoted its expression. D, Luciferase reporter assays indicated that miR‐613 directly targeted ANXA2. E, Western blot analysis results indicated that the upregulation of CYTOR reversed the expression of ANXA2, which was inhibited by miR‐613, whereas the downregulation of CYTOR decreased the expression level of ANXA2 promoted by miR‐613. *P < .05, **P < .01. Abbreviation: CYTOR, cytoskeleton regulator RNA

**Figure 8:**
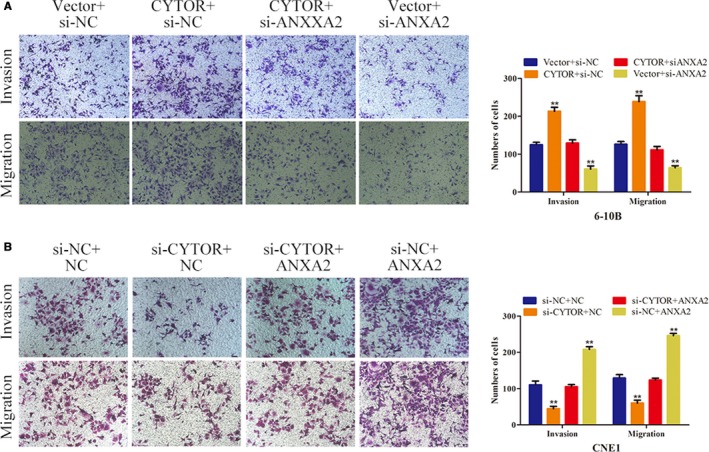
CYTOR promoted the invasion and migration of NPC cells via the miR‐613‐ANXA2 axis. A,B, The results of transwell assays showed that ANXA2 knockdown antagonized the positive effects of CYTOR overexpression, whereas ANXA2 overexpression overcame the inhibitory effects induced by CYTOR knockdown. *P < .05, **P < .01. Abbreviations: CYTOR, cytoskeleton regulator RNA; NPC, nasopharyngeal carcinoma

Then, we determined the target gene that was involved in the CYTOR/miR‐613 axis. Bioinformatics analyses indicated that ANXA2, which is known as a tumor‐promoting gene, served as the potential target of miR‐613.^13^, ^14^ Western blot assays were used to detect the expression of ANXA2 after the transfection of miR‐613 mimic in NPC cells. The results suggest that miR‐613 overexpression downregulated the ANXA2 expression level (Figure 7A). In addition, the luciferase assay confirmed that miR‐613 suppressed the luciferase activities of the 3′‐UTR of ANXA2 but showed no obvious influence on the luciferase activities of the mutant (Figure 7B,C). Subsequently, we aimed to evaluate whether CYTOR affected the expression level of ANXA2. The data revealed that the upregulation of CYTOR reversed the expression of ANXA2, which was inhibited by miR‐613, whereas the downregulation of CYTOR decreased the expression level of ANXA2 promoted by miR‐613 (Figure 7D,E). Transwell migration and invasion assays also confirmed that ANXA2 knockdown antagonized the positive effects caused by CYTOR overexpression, while ANXA2 overexpression overcame the inhibitory effects induced by CYTOR knockdown (Figure 8A,B).

## 4 DISCUSSION

NPC is one of the most common malignant tumors in southern China, and it tends to invade regional lymph nodes and distant metastases with an unclear mechanism.^1^, ^2^ Clinically, although concurrent chemoradiotherapy has attained a high cure rate in localized NPC, metastatic patients continue to have a poor prognosis with a median overall survival of 11‐22 months.^1^ Hence, the molecular mechanism underlying tumorigenicity, invasion, and metastasis of NPC should be determined.

Several vital lncRNAs, such as DANCR, MALAT1, and HOTAIR, have been identified as closely related to NPC progression.^15^, ^16^, ^17^ Recently, the aberrant expression of CYTOR has been reported in some types of cancers, including gastric cancer, hepatocellular carcinoma, and colon cancer, in which it may act as an oncogene.^18^, ^19^ However, the functional role and molecular mechanism of CYTOR in NPC remain vague. In this study, we proved that CYTOR expression was upregulated both in NPC tissues and cells. Functional assays revealed that CYTOR overexpression promoted the invasion and migration of NPC cells, while CYTOR knockdown caused suppressive effects on cell invasion and migration. Our established spontaneous lymph node metastasis model also confirmed that CYTOR promoted the NPC cell metastasis in vivo. However, we did not observe obvious effects of CYTOR on the capability of NPC cells to proliferate. These findings imply that CYTOR might work as a tumor promoter in NPC metastasis, but its regulation mechanisms still need to be elucidated.

lncRNAs could modulate gene expression at the posttranscriptional level by competitively sharing MREs with miRNAs.^10^ Wang et al^20^ reported that a TGF‐β‐associated lncRNA, named EPB41L4A‐AS2, suppresses tumor growth and metastasis by functioning as the sponge for miR‐301a‐5p. A study by Miao et al also showed that LINC00612 acted as a ceRNA for miR‐590 to promote bladder cancer cell proliferation and invasion.^21^ CYTOR could competitively inhibit several miRNAs in many cancers, such as miR‐139‐5p,^22^ miR‐153,^7^ and miR‐497,^23^ indicating that interacting with miRNAs might be the potential mechanism through which CYTOR drives NPC metastasis. Herein, we first found that subcellular localization of CYTOR mostly occurred in the cell cytoplasm instead of the nucleus, providing the foundation for the ceRNA hypothesis. Then, we used bioinformatic prediction to determine the potential miRNA that could interact with CYTOR. Among these candidate miRNAs, miR‐613 attracted our attention due to its tumor‐suppressive role in various cancers.^24^, ^25^, ^26^ Accordingly, RT‐PCR and luciferase reporter analyses verified that CYTOR was directly bound to miR‐613. Moreover, miR‐613 impaired the invasion and migration of NPC cells. Through the rescue experiment, our data revealed that CYTOR promoted NPC cell invasion and migration by repressing miR‐613. ANXA2 is upregulated in NPC tissues and promotes the proliferation, invasion, and migration of NPC cells. Here, we proved through RT‐PCR and luciferase reporter analyses that miR‐613 directly targeted ANXA2. Further investigation revealed that CYTOR induced NPC cell invasion and migration by functioning as the sponge for miR‐613 and activating the ANXA2 expression. Yue et al reported that CYTOR promotes the metastasis of colon cancer by interacting with β‐catenin and boosting β‐catenin nuclear translocation, which increases the expression of CYTOR.^9^ Wang et al also demonstrated that CYTOR, NCL, and Sam68 formed the complex, contributing to colorectal cancer development.^27^ Our findings promoted the understanding of the underlying mechanism of CYTOR in tumor progression.

In conclusion, we provided evidence that CYTOR is upregulated in NPC tissues and cells and facilitates the invasion and metastasis of NPC cells. Mechanistic analyses showed that CYTOR induced the upregulation of ANXA2 by competitively binding to miR‐613, thus leading to NPC metastasis. Our results highlight the importance of CYTOR in NPC development and provide new insights into the potential therapeutic target for the treatment of NPC.

## CONFLICT OF INTEREST

The authors declare no potential conflict of interest.

## Data Availability

I confirm that my article contains a Data Availability Statement even if no data is available (list of sample statements) unless my article type does not require one. I confirm that I have included a citation for available data in my references section, unless my article type is exempt.
